# The Barlow Sanatorium Association

**Published:** 1916-04

**Authors:** Walter C. Klotz


### CORRESPONDENCE.

This department is intended for the presentation of news and views not coming within the scope of other departments, and particularly to afford opportunity for discussion and criticism of views elsewhere expressed.

### The Barlow Sanatorium Association

Chavez Ravine Road, Los Angeles, Cal., March 16th, 1916.
Dr. A. L. Benedict,
Editor of the Buffalo Medical Journal,
Dear Doctor:—

I appreciated your notice and abstract of my article on "Kroenig's Isthmus" in the Boston Medical and Surgical Journal. I am sorry to have given the impression, however, that I was describing this ancient method as original or even recommending it, in fact the object of my study was to point out the limitation of Kroenig's method as it had been repeatedly mentioned as a very important diagnostic point, in spite of the understanding as to the anatomical difference. That this mistake in interpretating my conclusions is not your fault was evidenced by the discussion at the meeting of the National Association, when several members arose to **agree** with **me** in **emphasizing** the **importance** of mapping out the apical field, when I had been trying to point out the **unimportance** of this method.

Hoping you will understand that I should want to correct this wrong impression, I am,

Very truly yours,

DR. WALTER C. KLOTZ.

